# Medication error in anaesthesia and critical care: A cause for concern

**DOI:** 10.4103/0019-5049.65351

**Published:** 2010

**Authors:** Dilip Kothari, Suman Gupta, Chetan Sharma, Saroj Kothari

**Affiliations:** Department of Anaesthesiology, G. R. Medical College, Gwalior, Madhya Pradesh, India; 1 Pharmacology, G. R. Medical College, Gwalior, Madhya Pradesh, India

**Keywords:** Adverse drug event, drug error, medication error

## Abstract

Medication error is a major cause of morbidity and mortality in medical profession, and anaesthesia and critical care are no exception to it. Man, medicine, machine and modus operandi are the main contributory factors to it. In this review, incidence, types, risk factors and preventive measures of the medication errors are discussed in detail.

## INTRODUCTION

“The error of one moment becomes the sorrow of whole life”

- A Chinese proverb

The management of anaesthesia and critical patients has become safe with the advent of newer safe anaesthesia drugs, good quality equipments and high standards of monitoring, but the practice of poly-pharmacy, complex working conditions and involvement of multilevel medical and paramedical staff expose these areas to potentially life threatening medication error at some point of the treatment process.

Although majority of these errors are without any serious adverse outcome but some of them are associated with increased morbidity and mortality leading to prolonged hospital stay, high cost of treatment and potential for litigation.[1,2] The Institute of Medicine (IOM) report highlights that 44000 - 98000 patients die each year as a result of medical errors, a large portion of these being medication related.[3]

## INCIDENCE

Medication errors are common in health care system and reported to be the seventh most common cause of death overall.[4] A total of 2266 members of the Canadian Society of Anaesthesiologists were approached to find out the incidence of medication errors. Surprisingly 30% of them admitted to experience at least more than one error in their lifetime.[5] Japanese Society of Anaesthesiologists (JSA) investigated 27454 anaesthesia procedures over a period of 8 years (1999 – 2007). Out of total 233 incidences of medication error, 6.2% were clerical errors, hence they were not included in the study. Rest were either over-dose (25%), substitution error (23%) or omission error (21%).[6] A total of 89% of respondents in a survey of anaesthesiologists in New Zealand have admitted to made a drug administration error at some stage of their carrier.[7] In a retrospective review of 2000 anaesthetic procedures in Australia, 144 were found to be involved in wrong drug administration.[8] In an another study of 55426 cases in Norway, 63 (0.11%) cases of a drug error were found, out of which 3 cases were classified as serious.[9]

Among critically ill patients, the rate of medical error ranges from 1.2 to 947 errors per 1000 ICU days with a median of 106 errors per 1000 ICU days.[10,11] In intensive care although majority (40%) were of wrong infusion rate.[12] In another study, the incidence of adverse drug event (ADE) was found to be similar in medical ICU and coronary care unit of a tertiary care hospital (127.8 and 131.5 errors per 1000 patient days, respectively).[13]

All these reports are the tip of the iceberg as many cases are not reported due to various reasons like different population variation, clinical practice variation, lack of uniformity in definition, method of reporting and collection of data, fear of blaming and defamation among colleagues etc.[14]

## TYPES OF MEDICATION ERRORS

A medication error is any error in the medication process, whether there are adverse consequences or not [Table 1]. Errors can be divided into two groups according to the working system:[15] active failure and latent conditions. *Active failure* are unsafe acts committed by people who are in direct contact with the patient, slip and lapse are skill behaviour errors whereas mistakes are knowledge-based errors due to perception, judgement, inference or interpretation. *Latent conditions* are due to the reasons within the system and occurs when individuals make decisions that have unintended consequences in the future.[15,16]

**Table 1:** Definition of medication errors[16]

Medical error	The failure of a planned action to be completed as intended or the use of a wrong plan to achieve an aim
Medication error	Any error in the medication process, whether there are any adverse consequences or not
Adverse drug event	Any injury related to the use of a drug. Not all adverse drug events are caused by medical error or vice versa
Preventable ADE	Harm that could have been avoided through reasonable planning or proper execution of an action
Near miss	The occurrence of an error that did not result in harm
Slip	A failure to execute an action due to routine behaviour being misdirected
Lapse	A failure to execute an action due to lapse in memory and a routine behaviour being omitted

Alternatively, errors can be classified as error of omission or error of commission. *Errors of omission* are defined as failure to perform an appropriate action whereas *errors of commission* are defined as performing an inappropriate action.[17]

**Figure 1:**
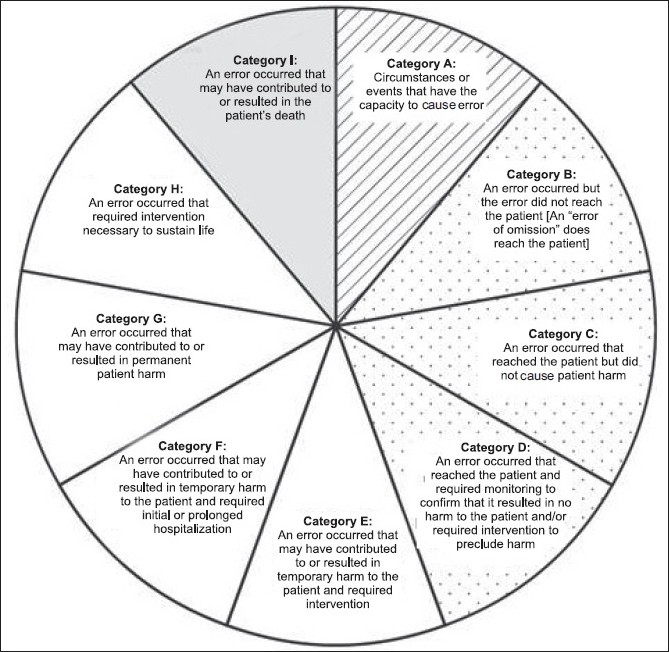
NCC MERP medical error index 18. No error: category A; error, no harm: category B, C, D; error, harm: category E, F, G, H; error, death : category I

The National Coordinating Council for Medication Error Reporting and Prevention (NCC MERP) realised the need for a standardised categorisation of error. On 16 July 1996, this council adopted a medication error index that classified the error according to the severity of the out come[18] [Figure 1]. This is required for categorising medication errors. The ISMP has already implemented this index for use in the database.

## TIME OF THE MEDICATION ERROR

Critical incidents occurred most commonly during middle of the anaesthesia (42%), frequently during induction (28%) and at the beginning of the procedure (17%).[19] Against the popular belief that most of the medication errors occur in late night shifts, Fasting *et al*[9] found that out of 63 drug errors only 1 occurred at night, whereas 56 incidences occurred in day shifts.

The administration of a single dose of any drug to the patient in critical care requires at least 80 - 200 individual steps at different stages namely prescription, transcription, preparation, dispersion, and administration.[20] Most errors occurs during the administration stage (53%), followed by prescription (17%), preparation (14%), and transcription (11%).[21]

## RISK FACTORS

Previous reviewers had concentrated primarily on quantitating the overall anaesthesia risk using mortality as the measure of negative outcomes. Human errors were believed to be a factor ranging from 65-87% for deaths during anaesthesia in several studies.[22–25]

Attempts to identify the risk factors have been limited to variables related to either surgical procedures or patients. Factors associated with anaesthesia and/or factors that may have predisposed to an anaesthesia error were not analysed. Furthermore, no study has focused on the process of error, devices, etc, regardless of the outcome. Cooper and colleagues[26] have identified several risk factors in a critical incident analysis to study preventable mistakes. Maximum errors were due to either inadequate experience (16%) or due to inadequate familiarity to equipment or device (9.3%) whereas haste and inattention or carelessness each amounted to 5.6% errors during anaesthesia [Table 2].

**Table 2:** Risk factors for errors during anaesthesia[26]

Inadequate total experience	77
Inadequate familiarity with equipment/device	45
Poor communication with team	27
Haste	26
Inattention/carelessness	26
Fatigue	24
Excessive dependency on other personnel	24
Failure to perform normal check	22
Training or experience	22
Lack of enough supervision	18
Environment or colleagues	18
Visual field restricted	17
Mental and physical factors	16
Inadequate familiarity with surgery	14
Distraction	13
Poor labelling of controls, drug	12
Supervision-related factors	12
Situation precluded normal precautions	10
Inadequate familiarity with the anaesthetic technique	10
Teaching activity underway	09
Apprehension	08
Emergency case	06
Demanding or difficult case	06
Boredom	05
Nature of activity related	05
Insufficient preparation	03
Slow procedure	03
Others	03
Total	481

Various other factors exist in operating rooms giving rise to a high incidence of medication errors during the conduction of anaesthesia. Lack of staff, overtime and odd working hours, inattention, poor communication, carelessness, haste and fatigue are the common factors related to medical and paramedical personnel.[8,19,27–29] Look-alike, sound-alike drugs,[30,31] confusing, inaccurate or incomplete drug labels and packaging,[31] swapping of syringe labels,[9,32] swapping of syringes and ampoules,[33] unlabelled syringe,[34] and failure of drug dose calculation,[5] have been reported in the literature from time to time.

In critical care, apart from those mentioned above, some other factors also exist which complicate the matter further. Severity of illness,[35] lack of usual medication list,[36] need of sedation and artificial ventilation,[37] calculation and programming of infusion pumps,[38] inexperience, lack of drug knowledge, sleep deprivation of provider,[11,39,40] high stress and fast pace of medication[10] and frequent changes in drugs and doses[41] have been listed by different authors.

## DRUGS INVOLVED IN MEDICATION ERRORS

Various group of drugs involved in medication errors during practice of anaesthesia have been reported by different authors. Induction agents like pentothal sodium, ketamine, depolarizing and non-depolarizing muscle relaxants, narcotic and sedatives, anticholinergics, and local anaesthetics have been given wrongly either due to misidentification, wrong labelling, syringe swap, or exchange with another drugs because of inattention or haste. However, in majority of the cases these errors did not result in any serious harm to the patients.[5,9,42]

In critical care units, the involvement of inotropes, narcotics, sedatives, analgesics, potassium chloride, magnesium sulphate, and anticoagulants like heparin or anti-infective agents have been identified in different studies.[12,13,43,44]

## CONSEQUENCES OF MEDICATION ERRORS

There is an increasing recognition that medication errors are causing a substantial global problem as many results in harm to patients and increased cost to health care providers, and anaesthesia and critical care are no exception to this.[45]

Medical errors are the leading cause of death in USA. A total of 44000 - 98000 Americans die every year. IOM has estimated that each year medical errors injured at least 1.5 million Americans and cost the health system more than 3.5 billion U.S. dollars. In another study approximately 7000 deaths in USA have cost more than 2 billion dollars.[3]

Medical errors erode not only a patient’s but also a family’s confidence in health care organisations, public confidence also suffers due to these errors.[46] The memory of errors can haunt the provider for years.[47] Anaesthesiologists have been charged for manslaughter, homicide, etc.[7,30,48]

## PREVENTION OF MEDICATION ERRORS IN ANAESTHESIA AND CRITICAL CARE

Very often anaesthesia professionals have been compared with commercial pilots because both face a high incidence of accidents either in take-off (induction of anaesthesia) and landing (emergence from anaesthesia) but this is not always true. Working in an operating room is much more complex than being in a commercial aviation set-up.[49] The aviation industry has adopted a definitive safety culture whereas anaesthesia professionals have a attitudinal barrier to safety. Both accidents and incidents in the aviation industry are taken as an opportunity to redesign the faulty system hence having a well developed feed-back and information system whereas an accident during the period of anaesthesia is often not reported due to fear of being blamed for carelessness, forgetfulness and sometimes character weakness.[50]

Most often, the number of people involved in single airline accident is much higher than that related to anaesthesia, hence airline accidents grab quick attention and response. Although deaths due to anaesthesia errors are much higher in number but they are sporadic, spread over a large span of time and invisible; therefore, they do not get immediate attention.[51]

Anaesthesiologists are one of the few groups of physicians who are personally responsible for drug administration. During anaesthesia most drug errors are totally or partially attributed to human error which is an inherent part of human psychology and activity; hence the occurrence of error can only be reduced and not eliminated.[52,53]

In general, following things should be kept in mind while working in the operation room to minimise the incidence of medication errors:

1. Reducing the complexity of the system to simple and linear to enhance the safety.[15,54]
2. Redundancy and standardisation are the basic principles in the design of a safe system.[15,54]
3. Double checking of ampoules, syringes and equipment before starting the procedure.[9]

Simple vigilance during the handling and administration of drugs is of utmost importance.

After a systemic review, Jenson and colleagues[55] recommended a 12-point strategy to prevent medication errors during anaesthesia and critical care:

1. The label on any drug ampoule or syringe should be read carefully before the drug is drawn up or injected.
2. Legibility and contents of labels on ampoules and syringes should be optimized according to agreed standards with respect to font, size, colour and information.
3. Syringes should always be labelled.
4. Formal organisation of drug drawers and work space should be used with attention to tidiness, position of ampoules and syringes, separation of look-alike drugs and removal of dangerous drugs from the operation room.
5. Labels should be checked specifically with the help of a second person or a device like bar code reader before administration.
6. Error during administration should be reported and reviewed.
7. Management of inventory should focus on minimising the risk of drug error.
8. Look-alike packaging and presentation of the drug should be avoided where possible.
9. Drug should be presented in prefilled syringes rather than ampoules.
10. Drug should be drawn up and labelled by the anaesthesia provider himself/herself.
11. Colour coding by class of drugs should be according to an agreed national or international standard.
12. Coding of syringe according to position or size should be done.

Several other measures to promote safe drug administration during anaesthesia and critical care have been suggested:-[33,56–58]

1. The provision of all labels in a standardised format emphasising the class and generic name of each drug incorporating the bar code and class-specific colour code as per international standard.
2. The use of a bar code reader to scan the drug at the point of administration immediately before it is given linked to an auditory prompt to facilitate checking of the drug identity.
3. Integration of scanned information into an automated anaesthesia record and reducing the cognitive load on the anaesthetist.
4. The use of devices at the point of care to automatically measure the dose of the administered drug.
5. A dosing nomograph on the infusion syringe label to avoid the need of look-up tables or dose calculations.
6. The automated medication dispensing system with features such as single issue drawers and bar code scanners to facilitate safer dispensing of drugs.

Camire *et al*[14] in a review article have suggested seven strategies to prevent errors in ICU. These are as follows:

- Eliminating extended physician work schedules.
- Computerised physician order entry.
- Implement support system for clinical decisions.
- Computerised intravenous devices.
- Active participation of pharmacists in ICU.
- Medication reconciliation.

Merali *et al*[59] made several recommendations to reduce medication errors at different stages of the system [Table 3].

**Table 3:** Recommendations to reduce medication errors[59]

Patient information	• Consistent documentation and complete operative medication history
	• Add prompts to pre admission card
Drug information	• Provide enhanced pharmacist support
Communication of drug orders and information	• Eliminate use of dangerous abbreviations and dose expressions
	• Incorporate computerised physician order entry into strategic planning
Drug labelling, packaging and nomenclature	• Enhance communication mechanism
	• Standardised anaesthetic cart trays and consider usage pattern
	• Labelling of all medication and solutions
	• Standardise labelling procedures
Drug standardisation, storage and distribution	• Evaluate the need and then clearly identify and segregate hazardous products
	• Increased provision of premixed solutions
	• Segregate and label, storage areas for neuromuscular blockers
	• Acquisition of prefilled automated dispensing cabinet
	• Incorporate bar coding system
Environment and workflow	• Minimize advance preparation of drug syringe
	• Return or remove unused medication from work cart
Staff competency and education	• Investigate, evaluate and educate staff about the dangers associated with workaround practices
Patient education	• Provide enhanced education material for preoperative patients
	• Consider pharmacy involvement in same day assessment
Quality processes and risk management	• Encourage reporting (including nearmisses) by all practitioners
	• Consider monitoring use of all trigger drugs
	• Consistently employ independent double checks for hospital selected ‘“high alert”’ drugs

Many organisations are now dedicated to patient safety, including IOM, Institute for Safe Medical Practice (ISMP), Emergency Care Research Institute (ECRI), Joint Commission for Food and Drug Administration (FDA), Centre for Medicated and Medicare Services (CMS), National Patient Safety Foundation (NPSF), United States Pharmacopeia (USP), Agency for Healthcare Research and Quality, National Quality Forum and many more.[60]

## CONCLUSION

Despite the best efforts, the increased use of technology and high standards of both invasive and non-invasive monitoring in anaesthesia and critical care, medication errors continue to occur even at the best centres worldwide. Simple vigilance, standardised protocol, and ‘think before act’ are the key factors to avoid occurrence of medication errors.
